# MediLinker: a blockchain-based decentralized health information management platform for patient-centric healthcare

**DOI:** 10.3389/fdata.2023.1146023

**Published:** 2023-06-22

**Authors:** John Robert Bautista, Daniel Toshio Harrell, Ladd Hanson, Eliel de Oliveira, Mustafa Abdul-Moheeth, Eric T. Meyer, Anjum Khurshid

**Affiliations:** ^1^School of Information, The University of Texas at Austin, Austin, TX, United States; ^2^Dell Medical School, The University of Texas at Austin, Austin, TX, United States; ^3^Department of Population Medicine, Harvard Pilgrim Health Care Institute, Boston, MA, United States; ^4^Information Technology Services, The University of Texas at Austin, Austin, TX, United States; ^5^Dell Seton Medical Center at The University of Texas, Austin, TX, United States

**Keywords:** blockchain, decentralization, health information management, MediLinker, patient identity

## Abstract

Patients' control over how their health information is stored has been an ongoing issue in health informatics. Currently, most patients' health information is stored in centralized but siloed health information systems of healthcare institutions, rarely connected to or interoperable with other institutions outside of their specific health system. This centralized approach to the storage of health information is susceptible to breaches, though it can be mitigated using technology that allows for decentralized access. One promising technology that offers the possibility of decentralization, data protection, and interoperability is blockchain. In 2019, our interdisciplinary team from the University of Texas at Austin's Dell Medical School, School of Information, Department of Electrical and Computer Engineering, and Information Technology Services developed MediLinker—a blockchain-based decentralized health information management platform for patient-centric healthcare. This paper provides an overview of MediLinker and outlines its ongoing and future development and implementation. Overall, this paper contributes insights into the opportunities and challenges in developing and implementing blockchain-based technologies in healthcare.

## Introduction

One way of ensuring quality healthcare is to provide patients with immediate access to and control of their health information. A barrier to achieving this is that patients often have limited access to and control over their health information since these data are stored and managed by health institutions where they previously received healthcare. To illustrate, a patient might have one record in hospital A and another record in hospital B, and a provider from hospital C might not be able to access both records from hospitals A and B because of the absence of a health information exchange system (Castillo et al., 2018). This limits transparency in healthcare services provided to patients and reduces the quality of care provided to them. Nonetheless, the persistent siloing of health information contributes to healthcare fragmentation and inefficient healthcare delivery (Kelly et al., 2019).

Although siloing of health information between institutions can be addressed by centralization, a common drawback of such a strategy is the increased vulnerability of health information to a data breach. A recent report shows that “hacking/IT incidents” is the most commonly reported type of data breach incident among health-related institutions in the US (HIPAA Journal, 2023). Considering the sensitive nature of health information and in light of the increasing prevalence of hacking incidents, it is crucial to ensure the safety and security of health information, especially when it is stored electronically and transferred from one entity (institutions or patients) to another.

Recently, there have been efforts in the healthcare industry and academia to leverage blockchain technology to address centralization and security issues in the storage and transfer of health information. Although popularized by its use case in the form of cryptocurrency transactions, blockchain can be used in various use case scenarios, one of which includes storing and transferring health information (Angraal et al., 2017). Since it is based on a decentralized approach, it is considered to be a viable solution to safely store and transfer health information between patients and healthcare providers (Kuo et al., 2017). In an attempt to prove the feasibility of this concept in practice, we have been working on a blockchain-based health information management application we have called MediLinker.

The goal of this paper is threefold. First, we provide a brief overview of blockchain in healthcare. Second, we discuss MediLinker's development and how it enhances access, control, and security of health information. Finally, we conclude the paper by presenting MediLinker's future in terms of its development and implementation.

## Blockchain in healthcare

Blockchain refers to an immutable distributed digital ledger that logs data entries in a decentralized manner without the need for entities to interact with a central trusted third party (Hasselgren et al., 2020). Although blockchain is key to the operationalization of Bitcoin and other cryptocurrencies, scholars have explored its potential in healthcare (Angraal et al., 2017; Hang et al., 2019; Ng et al., 2021). Using blockchain is beneficial in healthcare because its key characteristics (i.e., decentralized management, immutable audit trail, data provenance, robustness/availability, and security/privacy) can be used to further improve the security of health information (Kuo et al., 2017).

Several studies provide insights into the state of blockchain in healthcare. One review found that most studies conducted technical designs and demonstrations of blockchain in healthcare and very few conducted clinical translation studies (Ng et al., 2021). Likewise, Angraal et al. (2017) noted that proposals on implementing blockchain in healthcare are usually short-term and have focused on data validation, auditing, and authorization because of the potential barriers (e.g., privacy, compliance, and data storage) to storing live health information within the blockchain. Another review noted that integrating electronic health records with blockchain should consider security, scalability, governance, interoperability, and privacy (Mayer et al., 2020). Finally, one review noted that blockchain should be implemented in health information systems because of its ability to ensure data integrity, access control, data logging, data versioning, and nonrepudiation (Elangovan et al., 2022). In general, findings from previous works provide a backdrop for developing and implementing MediLinker.

## Overview of MediLinker

The 21st Century Cures Act of 2016 has mandated that patients' access to their medical data should be made easy by their healthcare providers (HealthIT, 2022). Although healthcare organizations have spent billions of dollars to upgrade their electronic health records (EHRs), patient access to their records remains convoluted and hindered due to federated healthcare systems. The distributed nature of blockchain technology can provide a trusted peer-to-peer network that can connect these federated healthcare providers centered around a decentralized patient identity. To date, practical implementations of blockchain for universal healthcare identity and EHR management remain elusive. Self-sovereign identity systems provide a decentralized identifier (DID) to establish peer-to-peer connections and verifiable credentials (VC) for sharing digital records (Tobin and Reed, 2017).

Since 2019, our multidisciplinary team of healthcare providers, software engineers, blockchain experts, and user experience experts at The University of Texas at Austin's (UT Austin) Dell Medical School (through the Khurshid Labs in the Department of Population Health), School of Information, Department of Electrical and Computer Engineering, and Information Technology Services has developed and conducted rigorous research on a blockchain-based self-sovereign identity solution and health information management application called MediLinker.

In general, MediLinker is an identity wallet for the issuing and sharing of VCs between patients and their healthcare providers using blockchain technology. By using MediLinker, patients can present VCs with their demographics, profile photo, and medication history. Each credential attribute follows the Fast Healthcare Interoperability Resource (FHIR) v4.0.1 standard to enable data liquidity and schematic interoperability between multiple EHR systems through a patient's care continuum.

To manage a patient's self-sovereign identity, we leveraged the Hyperledger Indy public permissioned blockchain framework to store the patient's decentralized identifiers and schemas for each credential type. The credentials are stored “off-ledger” in patient-controlled digital wallets. Hyperledger Aries is used as a middleware layer (API) to connect Hyperledger Indy with the digital wallets. Both Hyperledger Indy and Aries are developed by the Hyperledger Foundation and used by previous works to integrate blockchain in EHRs (Manoj et al., 2022; Abdelgalil and Mejri, 2023). The MediLinker system is hosted on Amazon Web Services (AWS) (Amazon Web Services, Inc. Seattle, WA) since it is HIPAA (Health Insurance Portability and Accountability Act) compliant, making it ready for future adoption in a clinical setting (for more details, see Harrell et al., 2022).

Patients access their MediLinker wallets with a smartphone application with biometric authentication, while clinic staff interacts with a Web application. With the MediLinker application, patients can establish secure connections with their healthcare providers (via QR code), and then create their credentials with attributes verified by the clinic staff using the patient's government-issued identity document or other physical cards. Once confirmed, the clinic staff can issue the credential to the patient's digital wallet, which can be sharable digitally with other participating institutions without physical documentation. Our research team rigorously evaluated and showed technical feasibility of MediLinker's framework and workflows toward improving the transition of care and sharing of credentials during simulated in-person and virtual sessions using synthetic patient data (Khurshid et al., 2021; Abdul-Moheeth et al., 2022).

Aside from realizing the potential of the 21st Century Cures Act by making it easier for patients to access their health information, MediLinker was developed to easily comply with the HIPAA security rule (National Institute of Standards and Technology, 2022) by leveraging blockchain to enhance the confidentiality, integrity, and availability of electronic health information. To a greater extent, this project aims to contribute to the United Nation's Sustainable Development Goal 3 (i.e., Good Health and Well-being) by demonstrating the use of blockchain as a means of leveraging digital transformations to sustainably improve health systems (Kickbusch et al., 2021).

## Development phases

**Figure 1:**
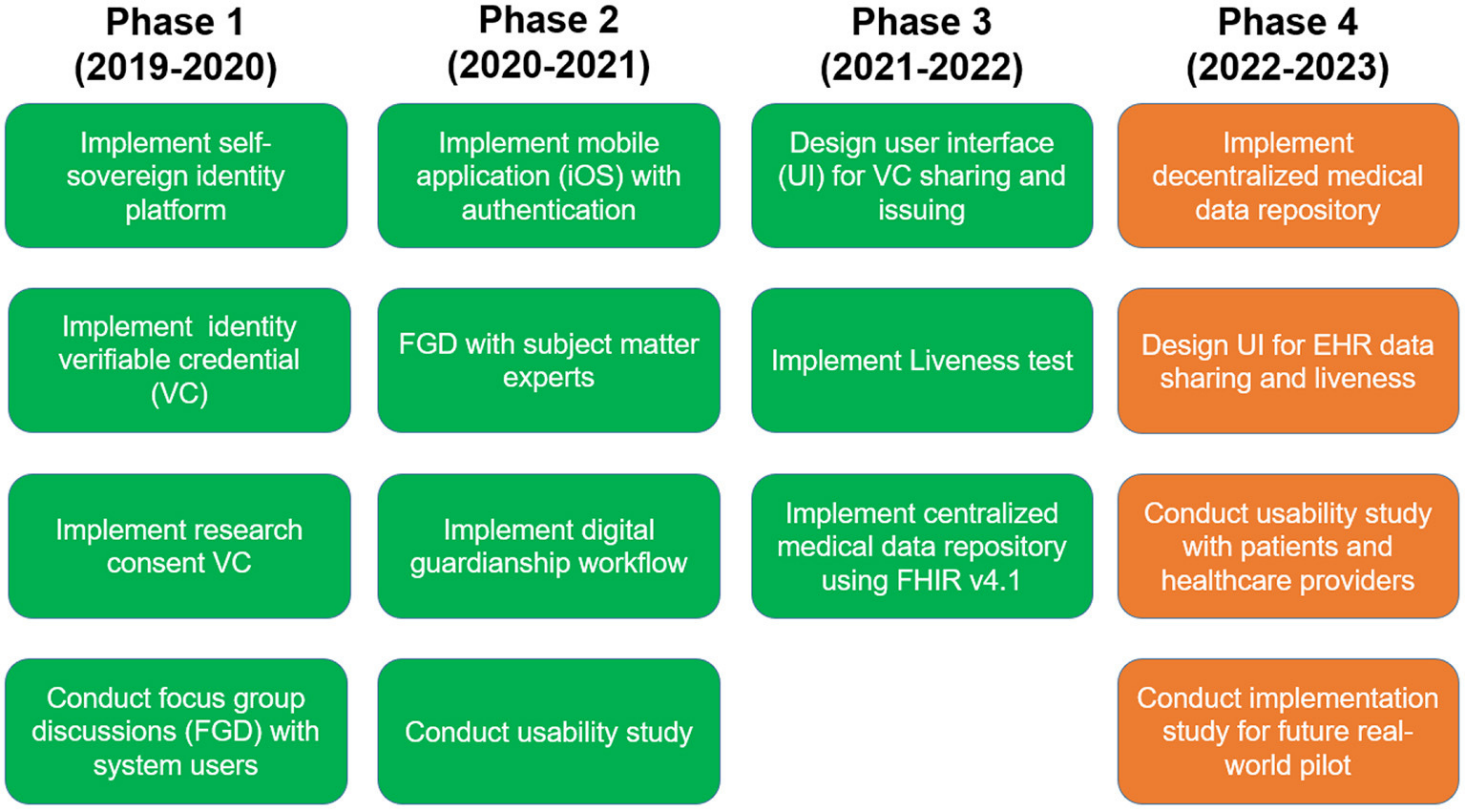
MediLinker phases.

Since starting in 2019, MediLinker has undergone several phases of research and development. Figure 1 summarizes key activities for Phase 1–4.

### Phase 1 and Phase 2

**Figure 2:**
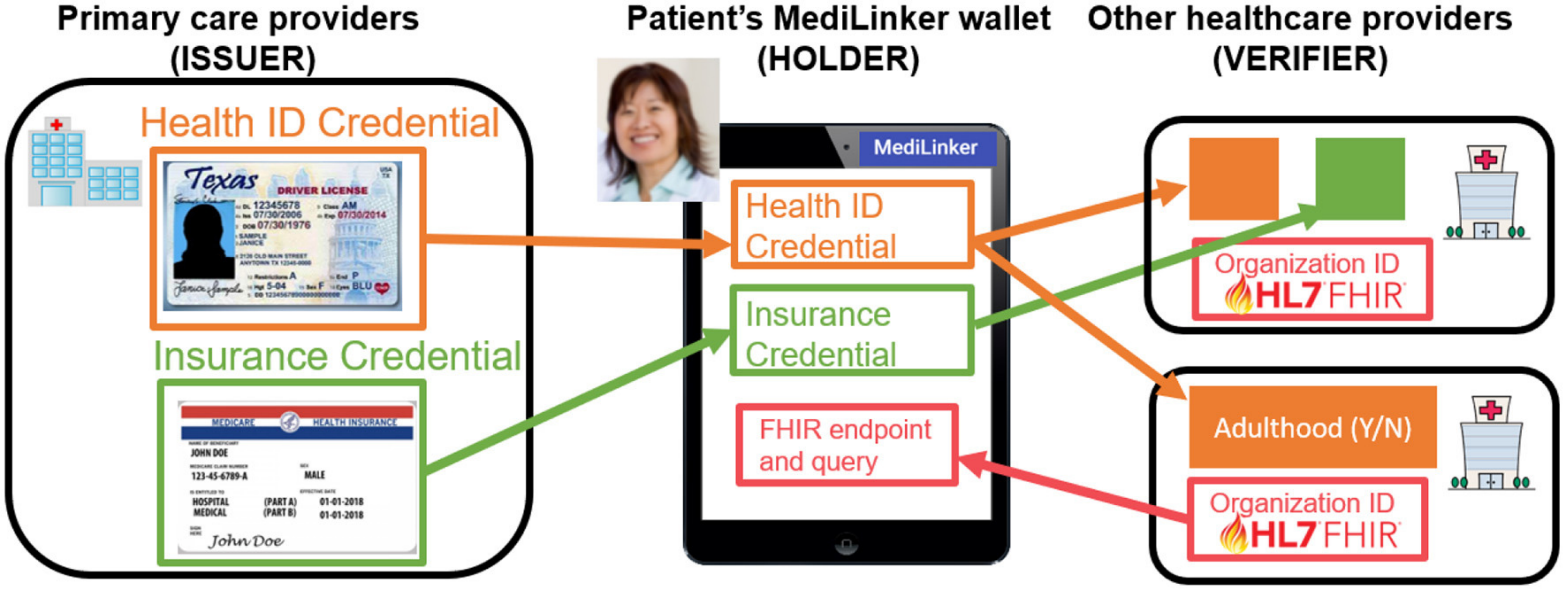
MediLinker manages the exchange of verifiable credentials.

In Phase 1 (2019–2020) and 2 (2020–2021), we established a proof of concept (POC) for patient-centric data sharing with a custom-built web application (Phase 1) and low-fidelity iOS application with biometric authentication (Phase 2). MediLinker provides patient and organization digital wallets that issue and manage VCs such as a Health ID for patient demographics and organizational IDs for clinics, banks, and insurance companies (see Figure 2).

In addition, patients can share their data, such as medication lists, research consent, credit card, and digital guardianship, with their healthcare providers (Harrell et al., 2022). Our results showed the feasibility of MediLinker's framework and workflows through simulated primary care clinic scenarios during in-person and virtual sessions using synthetic patient data (for more details, see Khurshid et al., 2021) and usability study with simulated patients (for more details, see Bautista et al., 2022a). More importantly, a focus group discussion with experts provided us with valuable insights on clinical (e.g., integration with existing clinical systems and adoption of clinicians), organizational and regulatory (e.g., accountability compliance, and legal safeguards), and ethical and social (e.g., trust, transparency, digital divide, health-related digital autonomy) issues when implementing MediLinker in clinical settings (for more details, see Bautista et al., 2022b).

### Phase 3

In Phase 3 (2021–2022), to transition MediLinker from a POC to a minimum viable product (MVP) for EHR data management, we implemented a data liquidity module for patient-controlled transmission of sensitive medical data with a high level of assurance using a liveness test. Specifically, the patient-controlled data is transferred between a trusted clinic's simulated EHR and a MediLinker medical data repository using the HL7 FHIR standards version 4.1. Our current implementation utilizes a health information exchange (HIE) model for MediLinker users. However, to provide patients with more control over their medical records, we will transition to a patient-centric HIE or HIE-of-One model (Gropper, 2016).

A liveness test using live video streaming was used to confirm that the patient is human and present in-person to provide high-security assurance of sensitive information. The patient starts the streaming from the MediLinker smartphone application. During the issuing of a Health ID credential, a patient's profile photo is taken and visually confirmed by the receptionist. In future clinical visits, the clinic staff can visually verify the patient's identity by comparing their face with the credentialed photo when increased assurance is required. In addition to the liveness test, we worked with UT Austin's School of Information researchers and student designers to improve the MediLinker application's user interface and user experience by developing workflows and conducting usability studies with volunteer testers.

### Phase 4

The project is currently in Phase 4 (2022–2023). The goal of this phase is to prepare the MVP version of MediLinker for implementation in primary care institutions since patients in this setting are known to frequently access their records before and after clinic visits (Zhong et al., 2018; Huang et al., 2022). Besides, recent works are primarily geared toward integrating blockchain in EHRs in tertiary settings (Hang et al., 2019; Lee et al., 2022; Mishra et al., 2022). Thus, publications resulting from Phase 4 will complement literature on the use of blockchain in other healthcare settings.

Initially, we compiled findings from our team's previous studies (e.g., Kelly et al., 2019; Abdul-Moheeth et al., 2022; Bautista et al., 2022a,b; Harrell et al., 2022) to guide the design and development of a fully functional MediLinker iOS application that could be used by patients and healthcare staff in primary care clinics. Moreover, we conducted a survey in August 2022 among 913 US adults (recruited via Amazon Mechanic Turk) to understand their willingness to use MediLinker, including factors that lead to potential adoption. Preliminary findings show that a majority of the respondents are willing to use it to store and manage their health information (77%), share health information with healthcare providers (79%), and provide consent for clinical research (78%). The survey also revealed that the perceived benefits provided by MediLinker outweigh the perceived risks when predicting respondents' willingness to use it. Collectively, the results of the survey will be useful in marketing MediLinker once a stable version is released to health consumers and healthcare organizations.

Currently, we are in the midst of a usability study with patients and healthcare staff (i.e., clinic staff, nurses, physicians, and health administrators). The goals of the usability study are to make adjustments to the enhanced MediLinker iOS app and identify issues in the clinical workflow when it is to be implemented in primary care clinics. We plan to complete data collection in mid-2023 and start data analysis by end of 2023.

## Future direction

After Phase 4, we plan to proceed with Phase 5 in early 2024 which involves implementing MediLinker in selected primary care clinics. Implementing MediLinker using live health information from real patients in primary care clinics would require detailed planning to ensure not only the validity of the study but, most importantly, the safety of patients and their records. Considering the complexity of implementing technologies in healthcare, it is crucial for us to work with colleagues who specialize in dissemination and implementation (D&I) science. Incorporating D&I principles in implementing MediLinker would enable us to rigorously test it in a real clinical setting and provide insights on how to replicate it in other clinics (Leppin et al., 2020). In general, the results of the implementation study would provide us with a benchmark on the usefulness of MediLinker in a real clinical environment.

Beyond Phase 5, we envision Phase 6 that will involve MediLinker's commercialization by leveraging the capital markets to support future developments and implementations in the real world. Through commercialization, we aim to acquire resources as well as business expertise for MediLinker's scalability and sustainability. For instance, since the MVP version is limited to Apple iOS users, future work will be geared toward developing an Android version. Moreover, considering that more patients and clinics will use MediLinker in the future, there is a need to scale up the required computing power to adequately and safely create patients' digital wallets and manage DIDs. Besides, scaling up MediLinker would also mean finding an additional user base.

## Conclusion

Blockchain has several use cases and implementing it in the healthcare field not only benefits patients and healthcare providers, but also health systems. This serves as a catalyst for the growing research interests to explore how blockchain can be used to address issues in security, transparency, liquidity, and privacy related to personal health information. In this paper, we shared our experience on how university researchers design, develop, and implement a blockchain-based health information management application, such as MediLinker. In general, our project is just one of the many efforts by industry and academic researchers to realize the value and usefulness of blockchain through continuous development and implementation. Similar to other health information technologies, implementing it in a clinical environment will be rife with challenges (e.g., privacy concerns, lack of trust in blockchain, and legal/regulatory compliance). However, we believe that working closely with multiple stakeholders (e.g., patients, healthcare providers, health administrators, and regulators) during its implementation would allow us to address the challenges posed by implementing blockchain technologies in a clinical environment.

## Data availability statement

The original contributions presented in the study are included in the article. Further inquiries can be directed to the corresponding authors.

## Ethics statement

The studies involving human participants were reviewed and approved by the Institutional Review Board of The University of Texas at Austin. The patients/participants provided their written informed consent to participate in this study.

## Author contributions

JB, DH, LH, EO, MA-M, EM, and AK contributed to the design, acquisition of data and its interpretation, drafting/revising the manuscript, and for final approval of the original and revised manuscript.
